# Overexpression of Galectin10 Predicts a Better Prognosis in Human Ovarian Cancer

**DOI:** 10.7150/jca.54595

**Published:** 2021-03-05

**Authors:** Wenxiao Jiang, Mandika Chetry, Shuya Pan, Longyi Wang, Xueqiong Zhu

**Affiliations:** Department of obstetrics and Gynecology, the Second Affiliated Hospital of Wenzhou Medical University, Wenzhou 325027, China.

**Keywords:** Galectins (LGALS), Kaplan-Meier Plotter, Ovarian cancer, Prognosis.

## Abstract

To explore the prognosis of Galectins (LGALS) expression on patients with ovarian cancer, the prognosis of LGALS members in ovarian cancer was retrieved and analyzed by using 'Kaplan-Meier plotter' database. The relation of LGALS to overall survival (OS) was evaluated according to histological subtypes, clinical stages and pathological grade. Quantitative real-time polymerase chain reaction and western blot were used to detect the mRNA and protein expression of LGALS in ovarian cancer and normal ovarian cells. Immunohistochemistry was applied to evaluate the different expression of LGALS between cancer and normal tissues. In total patients with ovarian cancer, LGALS4, LGALS8, LGALS10 and LGALS13 mRNA levels were related to a better OS, and LGALS1 to a worse OS. LGALS1 predicted a worse OS in women with serous, stages III+IV or grade II ovarian cancer. LGALS4 predicted a better OS in patients with endometrioid, stages I+II or grade III ovarian cancer. LGALS10 predicted a longer OS in females with serous, all stages, or grade III cancer. LGALS8 overexpression was related to a better OS in all stages. Notably, mRNA and protein expressions of LGALS4, LGALS10 and LGALS13 were decreased in cancer cells than those in normal cells (*P*<0.05). Additionally, the immunostaining score of LGALS8, LGALS10 and LGALS13 expression were lower but LGALS1 was higher in caner tissues than those in normal tissues (*P*<0.001). In conclusion, LGALS10 possibly is a valuable biomarker for predicting a favorable prognosis in patients with ovarian cancer, especially with serous, all stages and grade III cancer.

## Introduction

Ovarian cancer has been the most frequent gynecological cancer and the dominating cause of death from gynecological malignant tumors worldwide 1. Despite the modern therapeutic strategies such as concurrent chemotherapy, latest surgical techniques and targeted therapies, the mortality rate of ovarian cancer is still high because of the delayed diagnosis in advanced clinical stages, resistance to chemotherapy and metastasis within the peritoneal cavity 2. Almost 75% women will relapse within 2 years, even though most patients achieve complete remission after treatment 3. Thus, adequate understanding of the underlying mechanisms is essential to identify novel markers for disease progression in advanced ovarian cancer. Moreover, due to lack of techniques with high sensitivity and specificity to find ovarian cancer in early stage, it is urgently required further specific cancer biomarkers to predict the occurrence and prognosis of ovarian cancer.

Galectins (LGALS) are the S-type lectins bound to galactosidase-containing glycoproteins that have a conserved carbohydrate-recognition domain (CRD) with increasing attention as possible regulators in cellular physiology, such as invasion, inflammation, cell adhesion, migration, survival and synthesis 4. At present, there are 15 different types of LGALS identified in mammals and sub-classified with respective structural characteristics; proto-type (LGALS-1, -2, -5, -7, -10, -11, -13, -14 and -15) containing one CRD, tandem repeat type (LGALS -4, -6, -8, -9 and -12) containing two distinct CRD communicating with linker peptide and lastly, chimera type (LGALS-3) consisting of unusual proline and glycine rich short stretches fused onto the CRD 5. Evidence has illustrated their aberrant expression in various cancers such as astrocytoma, melanoma, and lung, bladder, uterine, prostate, kidney, breast, thyroid and ovary cancers 6. In recent years, cancer associated changes in protein glycosylation have emerged as promising markers for therapeutic targets in a great number of diseases. These endogenous glycan-binding proteins can influence immunosuppressive loops through co-opting relevant suppressive receptors, interference co-stimulatory pathways or regulate activation, survival of immune cells and differentiation 7. In particular, LGALS members have been reported to contribute to various cancer proliferation signaling, cell death resistance, evasion of immune surveillance, angiogenesis and activation of metastasis 5, 8, 9. In addition, aberrant expression of LGALS was frequently correlated with classical prognostic markers such as lymph node status, clinical stages and tumor grades 10. Of note, LGALS1 accumulation in peri-tumoral stroma has been found to induce cancer cell progression and increase chemoresistance in ovarian cancer 11, 12. Emerging evidence has identified LGALS as a potential biomarker for progression and prognosis in epithelial ovarian cancer 1, 13, 14. Therefore, diagnostic and prognostic relevancy of LGALS were emphasized, though conflicting data with regarding to certain type of LGALS and relative cancer have been published 15. Nevertheless, the prognostic and predictive functions of distinct individual LGALS expression in women with ovarian cancer remains unclear.

Hence, in the present research we performed comprehensive exploration on the prognostic role of LGALS family members in patients with ovarian cancer along with its relations with clinical stages, pathological grades, and histological subtypes. Furthermore, in order to select a more valuable prognostic biomarker for predicting the prognosis of patients with ovarian cancer, the different expression of LGALS members between ovarian cancer cell lines and normal ovarian cell lines was observed, and the different expression between ovarian cancer tissues and normal ovarian tissues was also explored.

## Materials and Method

### Database analysis

An online KM plotter (http://kmplot.com/analysis) 16 database was searched on May 23, 2019 to evaluate the relations of every LGALS member expression to OS of ovarian cancer patients. Recently, it has been recognized with a total of 54,675 genes that have been confirmed for breast cancer 16-18, ovarian cancer 19, 20, lung cancer 21, hepatic carcinoma 22. In our study, the gene expression and data about prognostic significance for patients with ovarian cancer (n=1,816) were downloaded from Gene expression Omnibus, The cancer Genome Atlas cancer, and Cancer Biomedical informatics Grid datasets 20. Additionally, we studied the several clinical characteristics, such as clinical stages, pathological grades and histological subtypes in patients with ovarian cancer. Shortly, fifteen LGALS members (LGALS1-15) were inserted into the above database to obtain Kaplan-Meier survival plots. The cut-off points expression in each LGALS member were extracted based on the gene mRNA expression with auto select best cutoff value among the eligible ovarian cancer samples. Eventually, LGALS expression was distinguished to 'low' and 'high' groups regarding the comparisons between expression values and established cutoffs.

### Cell lines and cell culture

The human ovarian cancer cell lines ES2, A2780, and human normal ovarian epithelial cell IOSE80 were kindly provided by Professor Tianfeng Chen (Jinan University, China). OVCAR-3 was purchased from American Type Culture Collection (ATCC). OVCAR-3 is a cell line derived from human ovarian epithelial adenocarcinoma, ES2 from ovarian clear cell carcinoma, and A2780 from ovarian undifferentiated carcinoma. A2780, ES2 and IOSE80 cell lines were cultured in DMEM medium (Gibco) plus 10% FBS (FBS, Gibco) in 5% CO2 atmosphere at 37°C, and OVCAR-3 cell was cultured in RPMI-1640 medium with 10% fetal bovine serum.

### RNA extraction and Quantitative real‐time PCR (qRT‐PCR)

Total RNA was isolated using the TRIzol reagent (Invitrogen, USA) according to the manufacturer′s instructions, and cDNAs were synthesized by reverse transcription. qRT‐PCR was conducted with the QuantiNova SYBR Green PCR Kit (QIAGEN, Germany) under the following reaction conditions: 94.0°C for 30 seconds, followed by 39 cycles of 94.0°C for 5 seconds and 60.0°C for 30 seconds. GAPDH was set as an internal control for gene quantification. The numbers of technical and biological replicates were at least three times for each gene with qRT-PCR analysis. The primers used in this study were displayed as following:

Human LGALS1:

Forward: 5′-TCTCTCTCGGGTGGAGTCTT-3′

Reverse: 5′-GAGATTCAGGTTGCTGGCGA-3′

Human LGALS4:

Forward: 5′-TCTCACAGGACCAGCCACTA-3′

Reverse: 5′-ATCCTGCCCAACCACAAAGT-3′

Human LGALS8:

Forward: 5′-CCTTGCACTTTCCGGCAATC-3′

Reverse: 5′-GGGGGAGGTGTGAGCTACTA-3′

Human LGALS10:

Forward: 5′-GCGACCACTTGCCTGTTTCT-3′

Reverse: 5′-CATGACCACACGACGACCA-3′

Human LGALS13:

Forward: 5′-AATGTCTTCTTTACCCGTGCCA-3′

Reverse: 5′-AGCTGTGGGTCATTGATAAAAGAGT-3′

Human GAPDH:

Forward: 5′-GACTCATGACCACAGTCCATGC-3′

Reverse: 5′-CAGGTCAGGTCCACCACT GA-3′

### Western blot analysis

Whole-cell extracts were lysed by lysis buffer, and quantified by the bicinchoninic acid (BCA) method. Protein samples (40 µg) were added to sodium dodecyl sulfate-polyacrylamide gel electrophoresis (SDS‐PAGE) and transferred onto the membranes of PVDF (Millipore). After blocking with 5% skim milk, blots were incubated with primary antibodies anti‐LGALS1 (1:500, mouse anti‐human, Santa Cruze), anti‐LGALS4 (1:5000, mouse anti‐human, Abcam), anti‐LGALS8 (1:5000, mouse anti‐human, Abcam), anti‐LGALS10 (1:10000, mouse anti‐human, Abcam), anti‐LGALS13 (1:10000, mouse anti‐human, Abcam) or β-tubulin (1:2000, mouse anti‐human, Cell Signaling Technology) at 4°C overnight. The blots were incubated with horseradish peroxidase (HRP)- conjugated secondary antibody at room temperature for 2h, and visualized with chemiluminescence reagent by a Tanon 4100 gel imaging system. The intensities of protein bands were quantified by densitometry analysis using NIH Image J software (Rockville, MD, USA). Experiments were performed for three times.

### Immunohistochemistry

Upon appropriate approval from the ethics committee at the Second Affiliated Hospital of Wenzhou Medical University, we constructed 10 ovarian cancer tissue specimens obtained from staging surgery or cytoreductive surgery performed for patients with ovarian cancer and 10 normal ovarian tissues samples from patients who receiving surgery because of other gynecological diseases. Inclusion criterion contains nonpregnant, nonbreastfeeding women older than 18 years of age. Immunohistochemistry was performed on the tissue sections (4 μm) from 10 formalin-fixed, paraffin-embedded ovarian tumor tissues and 10 normal ovarian tissues, which have been pathologically confirmed. After dehydrating in xylene and graded ethanol, the slides were incubated with 0.3% hydrogen peroxide and then blocked with 10% normal goat serum. Following these, the sections were incubated with primary antibodies anti‐LGALS1 (1:20, mouse anti‐human, Santa Cruze), anti‐LGALS4 (1:50, mouse anti‐human,Abcam), anti‐LGALS8 (1:100, mouse anti‐human, Abcam), anti‐LGALS10 (1:100, mouse anti‐human, Abcam) and anti‐LGALS13 (1:100, mouse anti‐human, Abcam) at 4°C overnight. Subsequently, the sections were incubated with secondary antibody, and then detected with 3'3-diaminobenzidine tetrahydrochloride (DAB) (1:50 dilution, GIBCO) staining and counterstained with hematoxylin. Positive and negative controls were set for each experiment.

The staining strength was observed and scored by two authors blindly depending on the positive cell percentage and positive cell staining density. The positive cell percentage was recorded as 0 score with 0% positively stained cells, 1 score with 1%-25% stained cells, 2 score with 26%-50% stained cells, 3 score with 51%-75% stained cells and 4 score with 76%-100% stained cells. The positive cell staining density was graded as 0 with no staining, 1 with light yellow staining, 2 with yellow staining, 3 with brown staining. Finally, the immunoreactivity score (IRS) was evaluated by multiplying the percentage of positively stained cells by the staining intensity (score ranged from 0 to 12). The average value from the two referees was used as the final score.

### Statistical analyses

Statistical analyses were performed using SPSS 17.0 software (SPSS, Chicago, USA). Data was expressed as the mean ± standard deviation of the mean (SD), and Student's *t*-test was used for group comparisons. Survival curves were plotted by the Kaplan-Meier method and compared by the log-rank test. Hazard ratio (HR), 95% confidence intervals (95% CI) and log rank *P* were calculated. *P* value of <0.05 was considered as statistical significance.

## Results

### Prognostic values of LGALS in total patients with ovarian cancer

**Figure 1:**
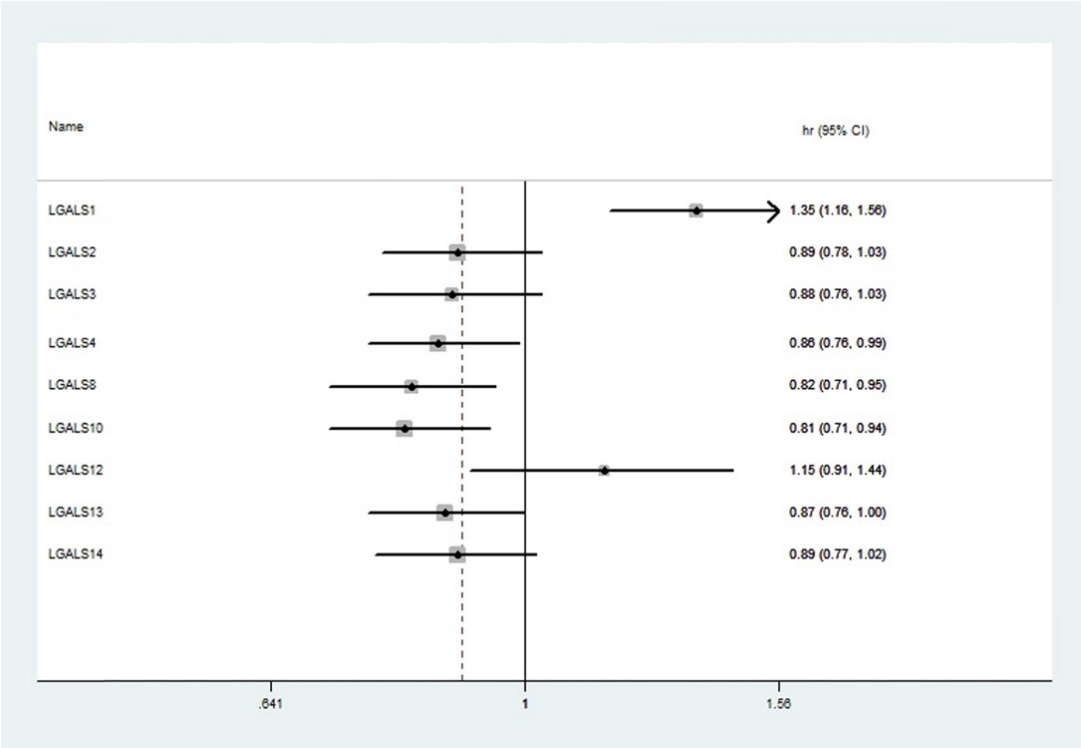
The prognostic HRs of individual LGALS members in all ovarian cancer in www.kmplot.com. HR: Hazard ratio; CI: confidence intervals.

**Figure 2:**
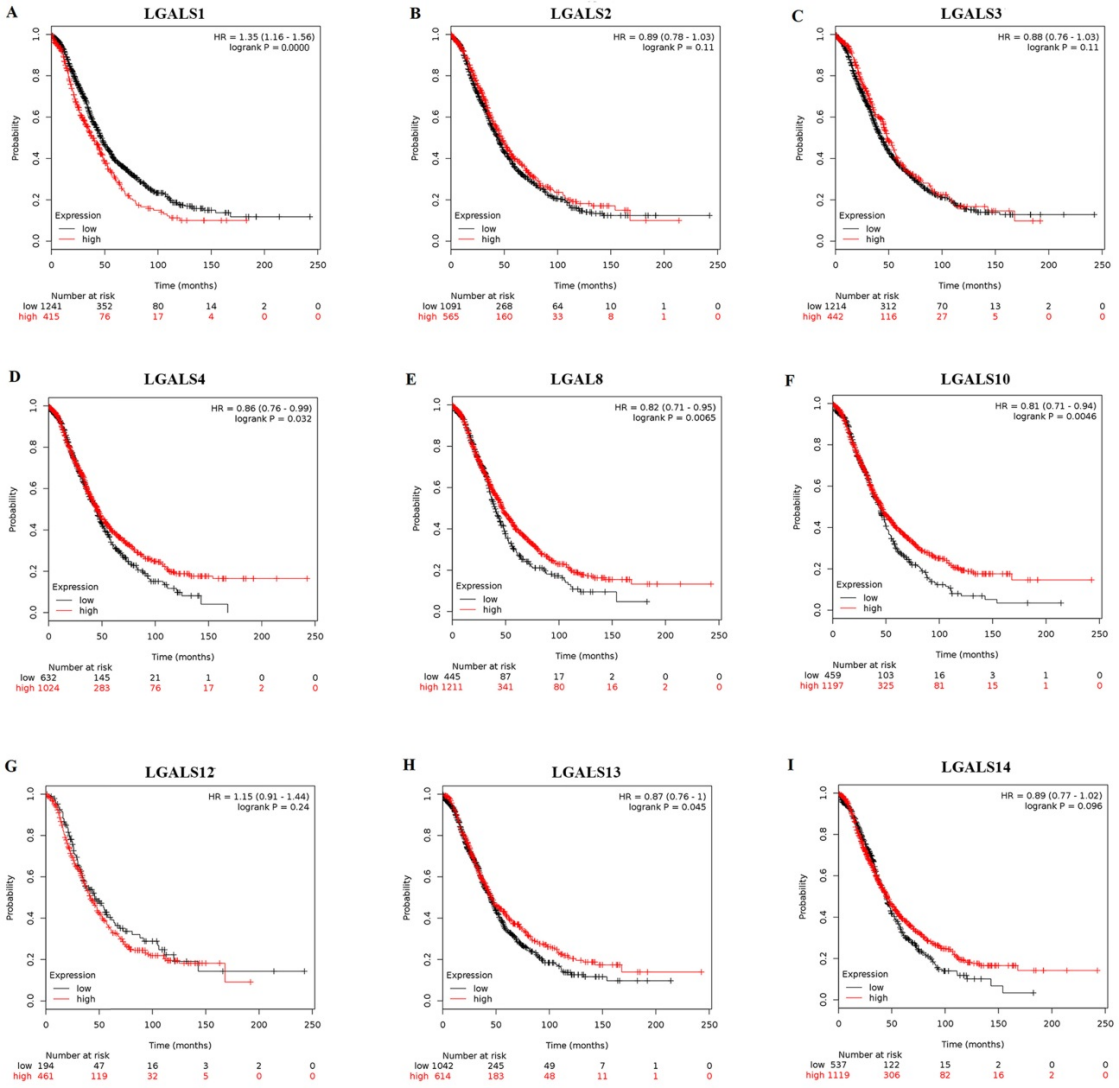
The prognostic value of LGALS members expression in ovarian cancer. The prognostic survival curves of LGALS1 **(A)** Affymetrix IDs: 201105_at), LGALS2 (**B**) Affymetrix IDs: 208450_at), LGALS3 (**C**) Affymetrix IDs: 208949_s_at), LGALS4 (**D**) Affymetrix IDs: 204272_at), LGALS8 (**E**) Affymetrix IDs: 210731_s_at), LGALS10 (**F**) Affymetrix IDs: 206207_at), LGALS12 (**G**) Affymetrix IDs: 223828_s_at), LGALS13 (**H**) Affymetrix IDs: 220440_at) and LGALS14 (**I**) Affymetrix IDs: 220158_at) were plotted for all ovarian cancer patients (n=655 for LGALS12 and n=1656 for the rest of LGALS members).

Among fifteen LGALS family members, merely nine members (LGALS1, LGALS2, LGALS3, LGALS4, LGALS8, LGALS10, LGALS12, LGALS13, and LGALS14) could be searched in http://www.kmplot.com, and no avalibale search data on other members can be found. Therefore, the nine LGALS members were studied and the prognostic values of their mRNA expression in patients with ovarian cancer were presented in** Figure 1.** Elevated mRNA expression of LGALS4, LGALS8, LGALS10 and LGALS13 was significantly related to a favorable OS in total patients with ovarian cancer, with HR=0.78 (0.69-0.89), *P*=0.0003; HR=0.86 (0.76-0.99), *P*=0.032; HR=0.82 (0.71-0.95), *P*=0.0065; HR=0.81 (0.71-0.94), *P*=0.0046; HR=0.87 (0.76-1), *P*=0.045. In contrast, elevated mRNA level of LGALS1 was significantly associated with a poor OS in total patients with ovarian cancer, with HR=1.35(1.16-1.56), *P <* 0.001. However, overexpression of LGALS2, LGALS3, LGALS12 and LGALS14 mRNA showed no correlation with OS in total patients with ovarian cancer, with HR=0.89 (0.78-1.03), *P*=0.11; HR=0.88 (0.76-1.03), *P*=0.11; HR=1.15 (0.91-1.44), *P*=0.24; HR=0.89 (0.77-1.02), *P*=0.096, respectively. These results were displayed in **Figure 2A-I**.

### Association between the prognostic values of LGALS1, LGALS4, LGLALS8, LGLALS10 and LGLALS13 in patients with ovarian cancer and clinicopathological parameters

Considering the significant correlation between LGALS level and survival outcomes of patients with ovarian cancer, the association between the prognostic values of LGALS1, LGALS4, LGALS8, LGALS10 and LGALS13 and clinicopathological features was further assessed, including tumor histological types, pathological grades and clinical stages (**Table 1**). Referring to different tumor histological types, among patients with serous ovarian cancer LGALS10 overexpression predicted a better OS and LGALS1 predicted a worse OS. For patients with endometrioid ovarian cancer, LGALS4 showed a favorable OS. However, LGALS8 and LGALS13 showed a null association with OS either in endometrioid ovarian cancer or in serous ovarian cancer. Furthermore, high expression of LGALS4 and LGALS10 mRNA in patients with grade III was related to a favorable OS, and elevated LGALS1 mRNA expression was correlated with a worse OS in patients with grade II. Nevertheless, high mRNA expression of LGALS8 and LGALS13 showed no correlation with OS among patients with any pathological grades. Regarding with different clinical stages of ovarian cancer, it was observed that elevated LGALS8 and LGALS10 mRNA expression predicted a better OS in patients with stages I+II as well as with stages III+IV, and LGALS4 predicted a better OS in patients with stages I+II. Meanwhile, LGALS1 expression presented a worse OS in patients with stages III+IV, but LGALS13 has no association with OS in patients with any stages.

### The different expression of LGALS1, LGALS4, LGALS8, LGALS10 and LGALS13 mRNA between ovarian cancer cells and normal ovarian cell

**Figure 3:**
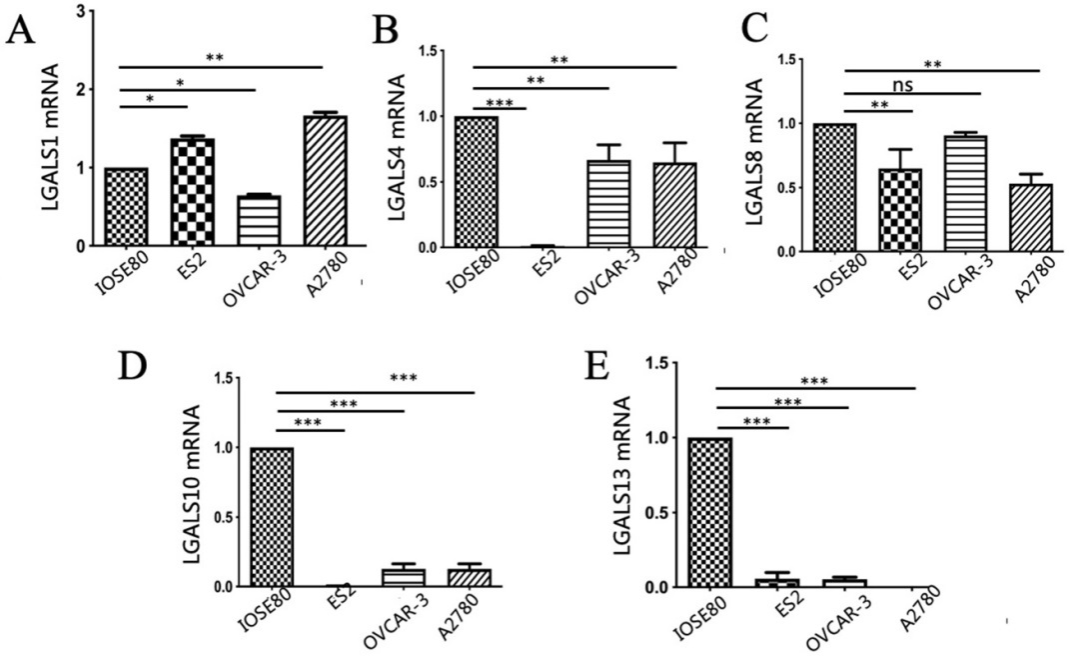
Analysis of** (A)** LGALS1, **(B)** LGALS4, **(C)** LGALS8, **(D)** LGALS10 and **(E)** LGALS13 mRNA expression levels in ovarian cancer cells and normal ovarian cell using qRT-PCR. * *P* < 0.01, ** *P* < 0.01, *** *P* < 0.001. ns: no significantly statistical difference.

As displayed in **Figure 3**, the mRNA expression of LGALS4, LGALS10 and LGALS13 were all significantly downregulated in the human ovarian cancer cell lines in comparison with those in normal ovarian cell line (*P* < 0.05). In addition, LGALS8 mRNA expression in ovarian cancer ES2 and A2780 cell lines was significantly lower than that in normal ovarian cell but its level in OVCAR-3 cell line showed no significant difference with that in normal ovarian cell (*P* = 0.14). In surprise, LGALS1 mRNA expression in normal ovarian cell was significantly lower than that in ovarian cancer ES2 and A2780 cell lines, but significantly higher than that in ovarian cancer OVCAR-3 cell line (*P* < 0.05).

### The protein expression of LGALS1, LGALS4, LGALS8, LGALS10 and LGALS13 in ovarian cancer cells and normal ovarian cell

**Figure 4:**
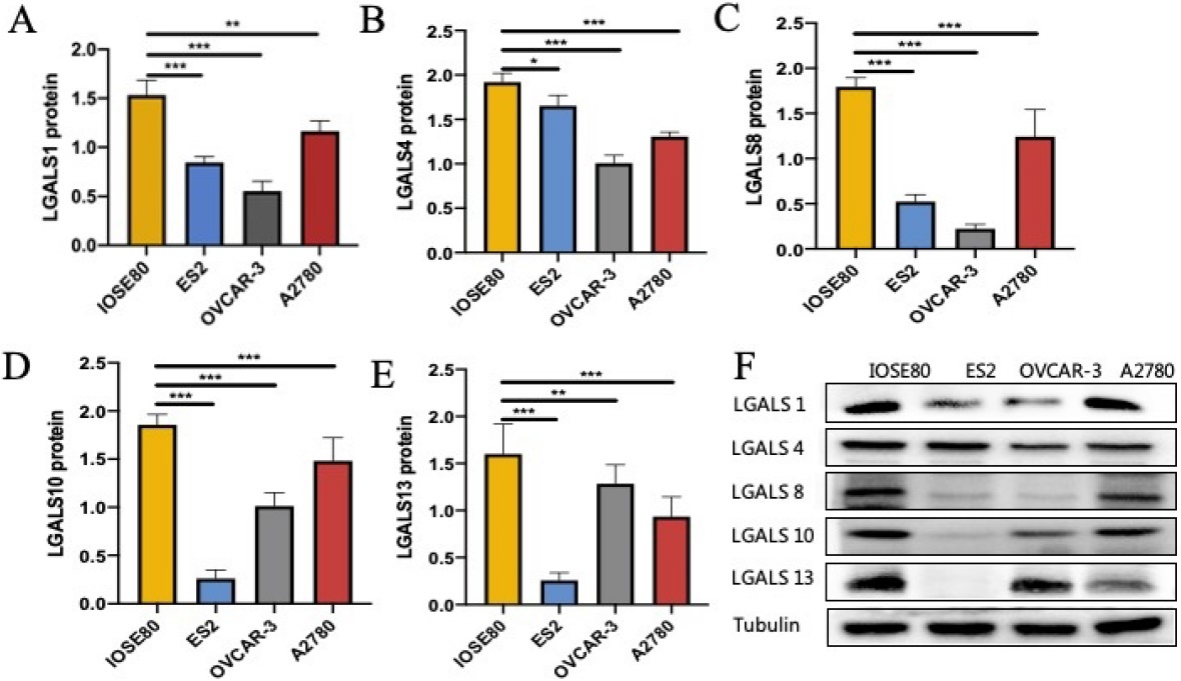
The protein expression of **(A)** LGALS1, **(B)** LGALS4, **(C)** LGALS8, **(D)** LGALS10 and **(E)** LGALS13 were detected in ovarian cancer cells and normal ovarian cell using western blot, and **(F)** showed the wetern blot bands. * *P* < 0.01, ** *P* < 0.01, *** *P* < 0.001.

As shown in **Figure 4**, the protein level of LGALS1, LGALS4, LGALS8, LGALS10 and LGALS13 in ovarian cancer cell lines were all decreased compared with those in the normal ovarian cell (all *P*<0.05).

### The different expression of LGALS1, LGALS4, LGALS8, LGALS10 and LGALS13 protein between ovarian cancer tissues and normal ovarian tissues

**Figure 5:**
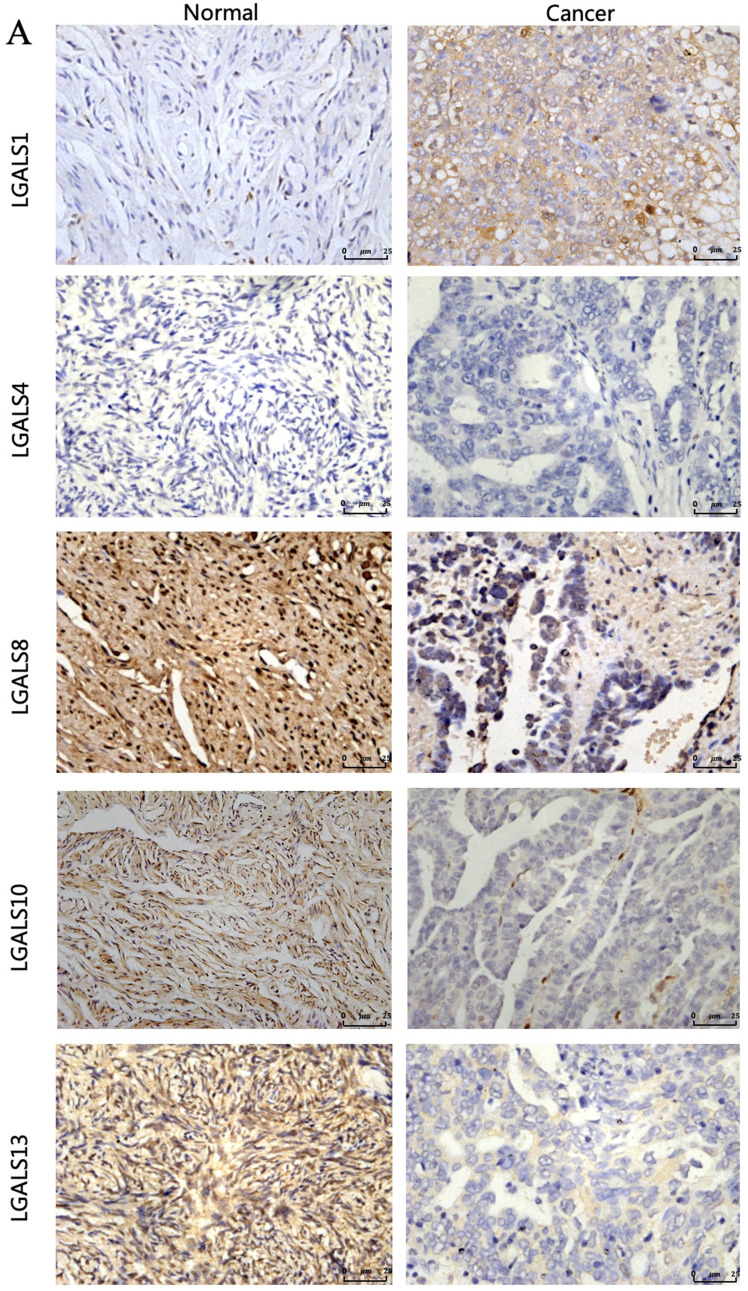
The representative protein expression of LGALS1, LGALS 4, LGALS 8, LGALS 10 and LGALS13 in human ovarian cancer tissues and normal ovarian tissues was detected by immunohistochemistry (SP staining, ×400).

The clinical characteristics of patients were displayed in **Table 2**. The immunohistochemistry results revealed that the staining of LGALS1, LGALS8, LGALS10 and LGALS13 were seen in the cytoplasm of positive cells, but no detectable immunostaining of LGALS4 in cytoplasm, nucleus or membrane was observed either in normal ovarian tissues or in ovarian cancer tissues analyzed (**Figure 5**). Notably, the staining score of LGALS8, LGALS10 and LGALS13 in ovarian cancer tissues were 3.04±1.24, 1.26± 0.65 and 1.52 ± 0.58, respectively, which were all lower than those in normal ovarian tissues (9.04±0.84, 7.24±1.69, 8.67±0.78) (all *P*<0.05). Conversely, LGALS1 protein expression in ovarian cancer tissues (8.45±0.63) was significantly higher than that in normal ovarian samples (2.02± 1.05) (*P*<0.001).

## Discussion

At present, the prognostic role of LGALS family in human cancer has been widely studied, however it is not clear about the roles of LGALS in ovarian cancer. By investigating the prognostic events of nine LGALS family members in patients with ovarian cancer, we found that elevated levels of LGALS4, LGALS8, LGALS10 and LGALS13 were related to a better OS in total patients with ovarian cancer, LGALS1 level was associated with a worse OS in total patients with ovarian cancer, and LGALS2, LGALS3, LGALS12 and LGALS14 had no significant prognostic influence on total patients with ovarian cancer. Hence, in the following discussion we mainly focused on the discussion of LGALS1, LGALS4, LGALS8, LGALS10 and LGALS13.

The predictive value of LGALS1 for tumor prognosis has been widely researched in multiple types of cancer. LGALS1 overexpression seems a poor prognostice factor for cancer. For instance, the survival rate of patients with gingival squamous cell carcinoma, lung cancer and colon cancer with strong LGALS1 expression was more significantly associated with a poorer outcome than that of patients with LGALS1 negative or weak expression 23-25. In our previous research, LGALS1 was uncovered to promote cell proliferation and inhibit cell apoptosis of cervical cancer 26. Additionally, LGALS1 has been suggested as an essential protein to lead malignancy in ovarian cancer cells through inducing cell proliferation and invasion, and also as a crucial chemotherapy (cisplatin) resistant factor 12. Chen et al. 1 revealed that patients with ovarian cancer with higher level of LGALS1 had increased recurrence in 3 years and poorer clinical outcomes compared with those with weak LGALS1 expression. Remarkedly, a recent report identified that high expression of LGALS1 in ovarian cancer cells was found in patients with higher histological grade, advanced stage and metastases 27. Consistently, we found that high level of LGALS1 was significantly related to a poor OS in total patients with ovarian cancer, especially with serous, stages III+IV and grade II cancer. However, no significant difference of LGALS1 expression between ovarian tumor cells and normal ovarian cell at mRNA level was observed, even though a higher protein expression of LGALS1 in ovarian cancer tissues and cells was identified compared with that in normal ovarian tissues and cells, respectively. We speculate that LGALS1 may influence the prognosis of ovarian tumors at the translation level.

LGALS4 has been identified as an adherent junction protein in porcine oral epithelial cells and primarily found in epithelial cells from the tongue to the large intestine but infrequently found in other human tissues 28. Recent evidence seemed to show an opposite efficacy of LGALS4 in prognostic value. In pancreatic adenocarcinoma, the increase of LGALS4 expression was significantly related to reduced lymph node and liver metastasis, and inhibited invasive and migratory behavior *in vitro* 29. Satelli et al. 30 exhibited that LGALS4 played a tumor suppressing role in colorectal cancer. Evidently, we showed that high expression of LGLAS4 was remarkably related to a favorable OS in total patients with ovarian cancer, especially with endometrioid, stages I+II and grade III ovarian cancer. Similarly, it was well demonstrated that LGLAS4 displayed a higher expression in normal ovarian cell compared with that in ovarian tumor cells at mRNA and protein levels. However, immunohistochemical study observed no staining of LGLAS4 in cancer tissues as well as in normal tissues. It may be explained by inactivation of antibodies, or the dewaxing of paraffin sections, which needs more researches in the future study.

Immunohistochemical study reported that LGALS8 expression was elevated in tumor tissues compared with that in normal tissues in bladder, kidney, lung, prostate and stomach 31. However, Wu et al. 32 defined that overexpression of intratumoral LGALS8 was a better prognostic factor for patients with gastric cancer. The reason for the conflict between the oncogenic function and better clinical survival outcome in gastric cancer possibly attributes to the dynamic process of LGALS8 during cancer progression. In the present study, however, we revealed that increased expression of LGALS8 mRNA predicted a favorable OS in all patients with ovarian cancer, as well as in patients with all stages. Similarly, our study validated a lower expression of LGALS8 in ovarian tumor cells and tissues than that in normal cell and tissues at protein level but not at mRNA level. It suggests that LGALS8 may influence the prognosis of ovarian tumors at the translation level. The different phenomeon in ovarian cancer may be due to that tumor microenvironment in ovarian caner likely has a promoting effect on the tumor suppressor function of LGALS8. In addition, the heterogeneity of methodologies also may be a related reason. However, the underlying functions and mechanisms of LGALS8 expression in ovarian tumors is still unclear and needs further research.

Moreover, multiple studies have suggested that LGALS10 was localized mostly in eosinophils and basophils 33, thus, its functions have been identified by its correlation with eosinophilic inflammatory diseases including asthma and allergic rhinitis 34-36. Reports on the role of LGALS10 in human cancers, however, are very few. Here, we confirmed that LGALS10 mRNA expression predicted a better OS in total patients with ovarian cancer, mostly in patient with serous, all clinical stages (I+II and III+IV) and grade III ovarian cancer through searching database. It implys that LGALS10 possibly exerts its predictive role in ovarian cancer. Specially, our study further identified the downregulation of LGALS10 in ovarian cancer cells compared with those in normal ovarian cell at mRNA and protein levels, and also showed the similar decreased expression in normal ovarian tissues compared with those in ovarian tumor tissues. It was speculated that LGALS10 may be a tumor suppressor in ovarian cancer, which needs more biological reserch. Overall, it suggests that LGALS10 may be an important marker in predicting a better prognosis for ovarian cancer patients.

Similarly, LGALS13 has been reviewed highly to have anti-inflammatory functions, which is uniquely expressed more in placental cells 37, and the decreased expression of LGALS13 in placental leads to preeclampsia. More implortantly, LGALS13 exerted the ability of promoting apoptosis of Th and Tc cell populations, and inducing the expression of CD25 and CD95 on T cells 38. However, the predictive role of LGALS13 in tumor especially in ovarian cancer is limited. In this study, we documented that elevated LGALS13 expression was related to a favorable OS in total patients with ovarian cancer, but has no association with OS in different tumor types, clinical stages or cancer grades. Additionally, this study showed the LGALS13 expression was lower in ovarian cancer cells and tissues than that in normal ovarian cell and tissues. LGALS13 might be an immunoregulatory molecule and provide an immunoprivileged environment to attack tumor. However, the role of LGALS13 for predicting prognosis of ovarian cancer still needs to be researched in the future mostly according to different clinicopathologic features such as tumor types, clinical stages and grades.

Our research was the first study to explore the prognostic fuanction of LGALS family for patients with ovarian cancer. However, there were some limitations in our study that needs to be considered. First, due to no avalibal data of 6 members (LGALS5, 6, 7, 9, 11, 15) in K-M plotter database among these fifteen LGALS members, we did not explore the predictive roles of these 6 LGALS members in ovarian cancer, although previous researches have widely studied their functions. For example, a meta-analysis demonstrated that overexpression of LGALS9 in cancer tissues was related to a longer cancer-specific survival (CSS) and OS in cancer patients 39. Secondly, the mechnisam by which LGALS10 was related to a better survival outcome and differently expressed in ovarian cancer and normal ovarian cells/tissues was not identified, which would be the research topic in our future study. Lastly, due to no fresh ovarian cancer or normal tissues collected, our study explore the different mRNA and protein expression of LGALS members only in cell lines but not in tissue samples. Instead, we performed immunohistochemistry with paraffin sections of tissues to detect the different expression of LGALS members, which need further study by using RT-PCR and western blot to explore the expression of LGALS in patients with ovarian cancer.

## Conclusion

In summary, our results showed that LGALS10 is related to a better OS and decreased in ovarian cancer cell and tissues, implying that possibly LGALS10 could be a favorable prognosis predicting marker for patients with ovarian cancer, especially in patients with serous, all clinical stages and grade III cancer. However, future research on biological mechanism is needed to validate the function of LGALS10.

## Figures and Tables

**Table 1:** Correlation of five LGALS gene expression with OS in different clinicopathological parameters in ovarian cancer patients

Clinicopathological parameters	LGALS		Cases	HR (95% CI)	*P*-value
Histology	LGALS1	Endometrioid cancer	37	331706418.13 (0 - Inf)	0.13
		Serous cancer	1207	1.31 (1.1 -1.56)	0.0024*
	LGALS4	Endometrioid cancer	37	0.07 (0.01 - 0.63)	0.0018*
		Serous cancer	1207	0.88 (0.76 - 1.03)	0.1
	LGALS8	Endometrioid cancer	37	4.06 (0.45-36.35)	0.17
		Serous cancer	1207	0.87 (0.74 - 1.02)	0.08
	LGALS10	Endometrioid cancer	37	318111595.97 (0 - Inf)	0.15
		Serous cancer	1207	0.84 (0.71 - 0.98)	0.028*
	LGALS13	Endometrioid cancer	37	3.96 (0.44-35.47)	0.18
		Serous cancer	1207	0.88 (0.74 - 1.05)	0.16
Pathological grades	LGALS1	I	56	2.16 (0.78 - 5.97)	0.13
		II	324	1.56 (1.12 - 2.17)	0.0087*
		III	1015	1.19 (0.98 - 1.45)	0.072
	LGALS4	I	56	1.85 (0.71 - 4.83)	0.2
		II	324	0.76 (0.55 - 1.05)	0.095
		III	1015	0.84 (0.71 - 0.99)	0.04*
	LGALS8	I	56	1.98 (0.75 - 5.18)	0.16
		II	324	0.74 (0.54 - 1.03)	0.074
		III	1015	0.9 (0.74 - 1.09)	0.27
	LGALS10	I	56	1.6 (0.63 - 4.07)	0.32
		II	324	0.75 (0.55 - 1.03)	0.077
		III	1015	0.83 (0.7 - 0.99)	0.035*
	LGALS13	I	56	1.96 (0.56 - 6.91)	0.28
		II	324	0.77 (0.56 - 1.06)	0.11
		III	1015	1.06 (0.9 - 1.25)	0.48
Clinical stages	LGALS1	I+II	135	2.15 (0.92 - 5.02)	0.072
		III+IV	1220	1.37 (1.16 - 1.62)	0.0003*
	LGALS4	I+II	135	0.37 (0.16 - 0.82)	0.011*
		III+IV	1220	0.86 (0.72 - 1.02)	0.08
	LGALS8	I+II	135	0.31 (0.14 - 0.67)	0.0017*
		III+IV	1220	0.79 (0.67 - 0.94)	0.006*
	LGALS10	I+II	135	0.32 (0.15 - 0.71)	0.0033*
		III+IV	1220	0.81 (0.69 - 0.95)	0.0079*
	LGALS13	I+II	135	0.58 (0.23 - 1.45)	0.24
		III+IV	1220	0.89 (0.76 - 1.05)	0.18

Notes: *P<0.05. **Abbreviations:** CI, confidence interval; HR, hazard ratio

**Table 2:** Clinical characteristics of ovarian cancer patients and control patients

Variables	Patients with ovarian cancer (N=10)	Patients with normal ovarian tissues (N=10)
**Median age (years)**	47 (range 34-55)	51 (range 37-60)
**Marital status, n (%)**		
Married	10 (100%)	10 (100%)
Unmarried	0	0
**Histology, n (%)**		
Serous	10 (90%)	─
Mucinous	0	─
Endometrioid	0	─
Clear cell	1 (10%)	─
**FIGO stage, n (%)**		
I+II	4 (40%)	─
III+IV	6 (60%)	─

## Abbreviations

CI: confidence interval
CRD: carbohydrate-recognition domain
HR: hazard ratio
KM plotter: Kaplan-Meier plotter
LGALS: Galectins
OS: overall survival
